# Whole-Exome Sequencing and Targeted Copy Number Analysis in Primary Ciliary Dyskinesia

**DOI:** 10.1534/g3.115.019851

**Published:** 2015-07-02

**Authors:** Christian R. Marshall, Stephen W. Scherer, Maimoona A. Zariwala, Lynette Lau, Tara A. Paton, Tracy Stockley, Rebekah K. Jobling, Peter N. Ray, Michael R. Knowles, FORGE Canada Consortium, David A. Hall, Sharon D. Dell, Raymond H. Kim

**Affiliations:** * Department of Pediatric Laboratory Medicine, The Hospital for Sick Children, Toronto, ON, M5G 1X8, Canada; † The Centre for Applied Genomics, Genetics and Genome Biology, The Hospital for Sick Children, Toronto, ON, M5G 1X8, Canada; ‡ Department of Molecular Genetics and the McLaughlin Centre, University of Toronto, Toronto, ON, Canada; § Department of Pathology and Laboratory Medicine, University of North Carolina School of Medicine, Chapel Hill, North Carolina; ** Division of Clinical and Metabolic Genetics, The Hospital for Sick Children, Toronto, ON, M5G 1X8, Canada; †† Department of Medicine, University of North Carolina School of Medicine, Chapel Hill, North Carolina; ‡‡ Division of Respirology, St. Michael’s Hospital, Toronto, ON, M5B 1W8, Canada; §§ Department of Medicine, University of Toronto, Toronto, ON, Canada; *** Child Health Evaluative Sciences, The Hospital for Sick Children, Toronto, ON, M5G 1X8, Canada; ††† Division of Respiratory Medicine, The Hospital for Sick Children, Toronto, ON, M5G 1X8, Canada; ‡‡‡ Department of Pediatrics, University of Toronto, Toronto, ON, Canada

**Keywords:** primary ciliary dyskinesia, whole-exome sequencing, copy number variation, diagnostic testing

## Abstract

Primary ciliary dyskinesia (PCD) is an autosomal-recessive disorder resulting from loss of normal ciliary function. Symptoms include neonatal respiratory distress, chronic sinusitis, bronchiectasis, *situs inversus*, and infertility. Clinical features may be subtle and highly variable, making the diagnosis of PCD challenging. The diagnosis can be confirmed with ciliary ultrastructure analysis and/or molecular genetic testing of 32 PCD-associated genes. However, because of this genetic heterogeneity, comprehensive molecular genetic testing is not considered the standard of care, and the most efficient molecular approach has yet to be elucidated. Here, we propose a cost-effective and time-efficient molecular genetic algorithm to solve cases of PCD. We conducted targeted copy number variation (CNV) analysis and/or whole-exome sequencing on 20 families (22 patients) from a subset of 45 families (52 patients) with a clinical diagnosis of PCD who did not have a molecular genetic diagnosis after Sanger sequencing of 12 PCD-associated genes. This combined molecular genetic approach led to the identification of 4 of 20 (20%) families with clinically significant CNVs and 7 of 20 (35%) families with biallelic pathogenic mutations in recently identified PCD genes, resulting in an increased molecular genetic diagnostic rate of 55% (11/20). In patients with a clinical diagnosis of PCD, whole-exome sequencing followed by targeted CNV analysis results in an overall molecular genetic yield of 76% (34/45).

Primary ciliary dyskinesia (PCD, MIM#244400) is a genetically heterogeneous, autosomal-recessive motile ciliopathy. Clinical manifestations include *situs inversus*, neonatal respiratory distress, progressive bronchiectasis, and respiratory failure in young adulthood. To date, there are 32 genes associated with PCD that account for a genetic diagnosis in approximately 65% of all patients with PCD (Kurkowiak *et al.* 2015).

The clinical diagnosis of PCD is challenging and relies on patient features and diagnostic investigations. These include diagnostic imaging, nasal nitric oxide levels, cilia beat frequency, and ciliary ultrastructure analysis by electron microscopy (EM) (Leigh *et al.* 2011). Historically, a diagnosis of PCD has relied on the findings of a classic ciliary ultrastructural defect on EM (Barbato *et al.* 2009). EM of normal motile cilia consist of nine peripheral microtubule doublets encircling a central microtubular complex supported by microtubular-associated proteins such as outer and inner dynein arms (ODA, IDA, respectively), radial spokes, and nexin links (Knowles *et al.* 2013a).

A molecular genetic diagnosis of PCD can be established through the identification of biallelic loss-of-function mutations in a PCD-associated gene. Mutations in certain PCD genes result in ciliary dysfunction and can give a specific ultrastructural defect on EM (Knowles *et al.* 2013a). ODA defects are the ultrastructural defect observed most frequently in PCD and are associated with mutations in *DNAH5*, *DNAI1*, *DNAI2*, *DNAL1*, *TXNDC3*, *CCDC114*, and *ARMC4* (Lobo *et al.* 2015). Defects in both ODA and IDA are seen in patients carrying mutations in *LRRC50/DNAAF1*, *KTU/DNAAF2*, *DNAAF3*, *CCDC103*, *HEATR2*, *LRRC6*, *ZMYND10*, *DYX1C1*, *C21orf59*, and *SPAG1*. IDA defects with microtubular disorganization are seen with mutations in *CCDC39* and *CCDC40*. Central microtubular abnormalities are observed with mutations in *RSPH1*, *RSPH4A*, and *RSPH9*. Patients with mutations in *CCNO* and *MCIDAS* have ciliary aplasia/oligoplasia and a marked reduction of cilia (oligocilia) due to a deficiency in ciliogenesis rather than motility. In addition, patients with mutations in *DNAH11*, *HYDIN*, *CCDC164/DRC1*, and *CCDC65/DRC2* do not have obvious ciliary ultrastructural defects and would be not be diagnosed with PCD on the basis of biopsy EM alone. We have shown previously that molecular genetic analysis can complement ciliary ultrastructure analysis and increase the overall diagnostic yield from 57% (28/49) to 69% (36/52) (Kim *et al.* 2014). However, the molecular genetics of PCD are complex and not considered routine in the diagnostic evaluation of patients (Barbato *et al.* 2009; Bush and Hogg 2012). Modern advances in molecular genetic technologies may overcome some of these perceived limitations in a cost-effective manner.

In genetically heterogeneous diseases such as PCD, selecting the appropriate gene and technique for molecular analysis is often difficult. Sanger sequencing is available for many, but not all, of the 32 PCD-associated genes. A step-wise approach by prioritizing genes based on ciliary ultrastructure and prevalence of mutations could be conducted using Sanger sequencing (Bush and Hogg 2012; Kim *et al.* 2014). However, as the number of genes tested increase, Sanger sequencing can become costly and time-consuming. In PCD, many new loci are being discovered on an ongoing basis, quickly out-dating existing available panels. Moreover, newly discovered loci account for a minority of patients with PCD (Knowles *et al.* 2013a), decreasing the diagnostic value for each gene added to a conventional panel.

Intron-exon level copy number variations (CNVs) are seen in up to 10.8% of autosomal-recessive Mendelian disorders and are not detectable by Sanger sequencing (Aradhya *et al.* 2012). Deletions in *SPAG*1, *ARMC4*, *DYX1C1*, *LRRC50*, and *ZYMD10* have been observed in isolated PCD cohorts (Loges *et al.* 2009; Blanchon *et al.* 2012; Hjeij *et al.* 2013; Knowles *et al.* 2013b; Tarkar *et al.* 2013; Zariwala *et al.* 2013), suggesting CNV analysis should be used in the molecular evaluation of patients with PCD. However, the prevalence and clinical significance of CNVs in other PCD-associated genes have not been examined.

Whole-exome sequencing (WES) has been used extensively for gene discovery and is beginning to be used in clinical practice in the diagnosis of genetically heterogeneous diseases (Neveling *et al.* 2013). The advantages of WES include providing a genomic approach in molecular genetic diagnoses through the analysis of all potential causative genes, including recently discovered genes not available on clinical gene panels (Boycott *et al.* 2015). Furthermore, this strategy allows for more complete interpretation of variants of uncertain significance (VUS), and can guide further molecular genetic analyses such as targeted CNV analysis. Because of ongoing gene discovery in genetically heterogeneous diseases such as PCD, WES may be a more cost-effective technique (Lucas *et al.* 2014; Kurkowiak *et al.* 2015). Here, we describe the complementary role of WES and targeted CNV analysis in solving cases of patients with PCD in a cost and time-effective manner.

## Material and Methods

### Study subjects

Between the period of August 2011 and December 2012, Sanger sequencing of 12 PCD genes was clinically available and conducted on 45 families with a clinical diagnosis of PCD (Kim *et al.* 2014). A total of 19 families (42%) were found to have biallelic pathogenic mutations in these 12 PCD genes and thus confirmed a molecular diagnosis. Pathogenic mutations were defined as mutations previously documented in patients with PCD; or nonsense, frameshift and splice-site mutations resulting in loss-of-function. Four families were solved in other research studies (Supporting Information, Table S1) leaving 22 families unsolved. Twenty families consented to have CNV analysis and/or WES. This study was approved by the Research Ethics Boards of the Hospital for Sick Children and St Michael’s Hospital. Study subjects provided written consent and/or assent where appropriate.

### Molecular genetic analysis algorithm

**Figure 1:**
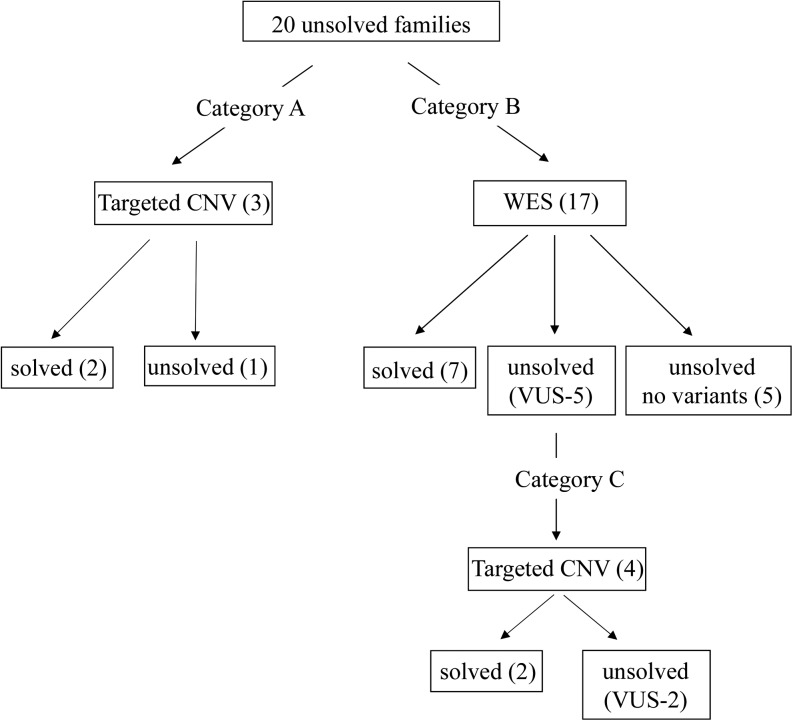
Molecular genetic analysis algorithm: study design and diagnostic yield per technology in patients with a clinical diagnosis of primary ciliary dyskinesia (PCD), defined as a ciliary ultrastructure defect and/or clinical features with low nasal nitric oxide (Kim *et al.* 2014). Sanger sequencing of 12 PCD genes provided a molecular diagnosis in 19 of 45 families. Of the 26 families who remained unsolved, 20 underwent targeted copy number variation (CNV) analysis and/or whole-exome sequencing (WES). Category A families had single pathogenic mutations in PCD genes after Sanger sequencing and underwent targeted CNV analysis alone to ascertain the second allele. Category B families had no pathogenic mutations after Sanger sequencing and underwent WES. Category C families had rare variants of uncertain significance after WES and underwent further targeted CNV analysis to ascertain the second allele. Four families had clinically significant CNVs and seven families were solved with WES alone giving an overall solved rate of 55% (11/20). These 11 families when taken together with 19 families solved through Sanger sequencing, and 4 families solved in other research studies, resulted in a solved rate of 76% (34/45).

A molecular genetic analysis algorithm was developed to maximize previous molecular genetic data and minimize time and cost. Individuals were divided into three categories depending on the information from previous Sanger sequencing and WES performed in this study (Figure 1). For the individuals who harbored single pathogenic mutations after Sanger sequencing, targeted CNV analysis of the putative causative gene was conducted to ascertain the second allele (category A). Individuals with no pathogenic mutations after Sanger sequencing underwent WES to analyze the remaining 20 genes not covered by Sanger sequencing (category B). WES-sequenced individuals who were not found to have any pathogenic mutations in a PCD gene, further analysis of missense variants occurring at a minor allele frequency of <1% was conducted. Rare missense variants were considered to be contributory if predicted to be pathogenic, disease causing or damaging by *in silico* prediction programs (MutationTaster, PolyPhen-2, SIFT). These individuals underwent further targeted CNV analysis on the suspected PCD gene (category C).

### Targeted CNV analysis

The selected technique for targeted CNV analysis was based on previously reported intron−exon CNVs in the literature. Exon 7 deletions in *DYX1C1* have been documented in other PCD cohorts (Tarkar *et al.* 2013), and a custom TaqMan copy number assay was designed to detect this specific CNV (File S1). Reported deletions of exon 62 of *DNAH5* (Berg *et al.* 2011) along with the remaining 78 exons were assayed using a commercially available, high-density gene-centric array comparative genomic hybridization (CGH; Prevention Genetics, Marshfield, WI). Similarly for *DNAH11*, where intron−exon level CNVs have not been reported, CGH was used to assay all 82 exons.

### WES and validation

WES was completed with the Illumina Hisequation 2500 platform at The Centre for Applied Genomics (TCAG) at the Hospital for Sick Children following whole-exome capture with the Agilent SureSelectXT Human All Exon V4 capture kit. Sequence reads were aligned to the reference human genome (hg19) with Burrows-Wheelchair Aligner 0.5.9. MarkDuplicates (Picard tools, version 1.79; http://broadinstitute.github.io/picard/) was used to remove any duplicate paired-end reads. Duplicate-free alignments were refined using base space local realignment and quality score recalibration using GATK 1.128. Mean depth of coverage was 138X (range 100−168X) with all cases having >95% of targeted bases sequenced to a depth of greater than 20X.

Single-nucleotide variants (SNVs) and insertion/deletions (indels) were called with default parameter in GATK 1.1.28. SNVs and indels were annotated using SNPEff (http://snpeff.sourceforge.net/) and ANNOVAR and filtered to differentiate novel variants from known polymorphisms by screening against public single nucleotide polymorphism databases (dbSNP, http://www.ncbi.nlm.nih.gov/projects/SNP/; 1000 genomes, www.1000genomes.org; NHLBI Exome Sequencing Project (ESP) Exome Variant Server http://evs.gs.washington.edu/EVS/) and our own internal database consisting of 283 exomes analyzed in the same manner at TCAG. Novel SNVs also were annotated with SIFT, PolyPhen-2, and MutationTaster to assess the putative variant effect on the proteins. SNVs were prioritized based on loss of function mutations (nonsense, frameshift, splice sites) and damaging missense mutations that fit an autosomal recessive disease model.

An *in silico* gene panel was created by filtered analysis of WES data. WES data were reanalyzed with the publication of new PCD genes and the *in silico* panel expanded in real-time. At the time of manuscript submission, 32 genes were analyzed for variants (*DNAH5*, *DNAI1*, *CCDC39*, *CCDC40*, *DNAI2*, *DNAH11*, *RSPH9*, *RSPH4A*, *KTU/DNAAF2*, *LRRC50/DNAAF1*, *TXNDC3/NME8*, *DNAL1*, *DNAAF3*, *CCDC103*, *HEATR2*, *HYDIN*, *LRRC6*, *CCDC114*, *CCDC164/DRC1*, *ARMC4*, *DYX1C1*, *ZMYND10*, *CCDC65/DRC2*, *RSPH1*, *OFD1*, *RPGR*, *C21orf59*, *SPAG1*, *DNAH8*, *CCDC151*, *MCIDAS*, and *CCNO)*. WES coverage of these genes was adequate with >98% of targeted bases sequenced and 92.08% (616/669) of exons having a mean coverage of >20X. Confirmation of variants was conducted on probands and available family members using standard Sanger sequencing methods in a CLIA-CAP diagnostic laboratory and genetic counseling was provided based on these results (Table S2).

## Results

Four individuals from three families harbored one pathogenic mutation after Sanger sequencing (category A). Two families (111, 113) each harbored two different nonsense mutations in *DNAH5* and CNV analysis of *DNAH5* was conducted using array CGH to ascertain the second allele. Two clinically significant CNVs in *DNAH5* were found and likely causative second allele, corresponding to the ODA defect observed on ciliary EM (Table 1). The third family harbored a previously reported nonsense mutation in *DNAH11*, c.8698C > T (p.R2900*) (Lucas *et al.* 2012). However, *DNAH11* CNV analysis with array CGH did not detect a CNV and this case remained unsolved.

**Table 1:** Biallelic pathogenic variants in known PCD genes through CNV and WES analysis

Family	Patient	Ethnicity	Sex	Dx Age	Situs Status	Ciliary EM	nNO nL/min	Gene	hg 19 Genomic Coordinates	Transcript	Exon/ Intron	Base Changes	Predicted Effect	Segregation
Compound Heterozygous by CNV														
111	4	White	M	4 yr	I	ODA	8.1	*DNAH5*	Chr5: 13701398	NM_001369.2	Ex 77	**c.13486C > T**	p.R4496* (1)	Unknown
								*DNAH5*	Chr5: 13816665-	NM_001369.2	Int 42	Ex 41-42 4.4kB del		Unknown
									13821065		Int 40			
113	12	Portuguese	F	6 yr	A	ODA	22	*DNAH5*	Chr5: 13865895	NM_001369.2	Ex 27	c.4237C > T	p.Q1413*	Maternal
								*DNAH5*	Chr5: 13931161-	NM_001369.2	Int 2	Ex 2 1.6kB del		Paternal
									13932789		Int 1			
113	36	Portuguese	M	14 yr	S	ODA	8.9	*DNAH5*	Chr5: 13865895	NM_001369.2	Ex 27	c.4237C > T	p.Q1413*	Maternal
								*DNAH5*	Chr5: 13931161-	NM_001369.2	Int 2	Ex 2 1.6kB del		Paternal
									13932789		Int 1			
Homozygous by WES														
115	14	East Indian	M	7 yr	S	Inadequate	37.5	*LRRC6*	Chr8: 133645009	NM_012472.4	Ex 5	**c.630delG**	p.W210Cfs*12 (2)	Paternal
115	26	East Indian	M	9 yr	S	ODA+IDA	5.8	*LRRC6*	Chr8: 133645009	NM_012472.4	Ex 5	**c.630delG**	p.W210Cfs*12 (2)	Maternal
141	50	Pakistani	F	5 yr	I	Inconclusive	17.1	*SPAG1*	Chr8: 101226130	NM_003114.4	Ex 12	c.1519dupA	p.I507Nfs*5	Unknown
140	49	Portuguese	F	18 yr	I	Not done	6.9	*ARMC4*	Chr10: 28276450	NM_018076.2	Ex 3	c.247G > T	p.E83*	Unknown
									Chr10: 28151418	NM_018076.2	Ex 18	c.2744del	p.L915*	Unknown
135	39	Pakistani	F	12 yr	S	Oligocilia	22.1	*CCNO*	Chr5: 54527295	NM_021147.3	Ex 3	**c.961C > T**	p.Q321* (3)	*Trans*
138	46	White	F	35 yr	S	Normal	53.2	*HYDIN*	Chr16: 71008480	NM_001270974.1	Ex 32	c.4866del	p.P1623Qfs*20	Unknown
									Chr16: 70866971	NM_001270974.1	Int 79	c.13680-1G > T	splice site	Unknown
Compound Heterozygous by WES														
120	24	White	M	2 mo	I	ODA+IDA	11.2	*ZMYND10*	Chr3: 50382964	NM_015896.2	Ex 1	**c.47T > G**	**p.V16G (4)**	Maternal
									Chr3: 50381183	NM_015896.2	Ex 3	**c.300del**	p.F101Sfs*38 (2)	Unknown
137	45	Hispanic	F	30 yr	S	Inadequate	17.1	*RSPH1*	Chr21: 43913159	NM_080860.3	Ex 2	c.85G > T	p.E29*	Unknown
									Chr21: 43906558	NM_080860.3	Ex 4	c.287dup	p.N96Kfs*2 **Knowles (5)**	Child carrier

Genomic coordinates are approximate for copy number variations. Further clinical characteristics previously described (Kim *et al.* 2014). Previously described mutations are in **bold** (1) (Hornef *et al.* 2006); (2) (Zariwala *et al.* 2013); (3) (Wallmeier *et al.* 2014); (4) (Moore *et al.* 2013); (5) (Knowles *et al*. 2014). PCD, primary ciliary dyskinesia; CNV, copy number variation; WES, whole-exome sequencing; Dx, diagnosis; EM, electron microscopy; nNO, nasal nitric oxide; M, male; I, *situs inversus*; ODA, outer dynein arms; F, female; A, *situs ambiguous*; S, *situs solitus*; inadequate = inadequate ciliated cells to analyze, IDA, inner dynein arms inconclusive EM; inconclusive = adequate sample.

A total of 18 individuals from 17 families who did not have any pathogenic mutations detected on initial Sanger sequencing (category B and C) underwent WES. Seven category B families were solved by WES alone where two pathogenic variants were found in one of the additional 20 PCD genes not analyzed by Sanger sequencing (Table 1). Molecular genetic results were congruent with ultrastructural analysis when available. For those cases with available family members for segregation analysis, phase was determined to be *trans*.

WES on individual 46 revealed a splice site (c.13680-1G > T) and frameshift mutation, c.4866del (p.P1623Qfs*20) in *HYDIN*. Because of the presence of a pseudogene on chromosome 1 (*HYDIN2*), further characterization of both variants was conducted using Sanger sequencing (Olbrich *et al.* 2012) (File S1 and Figure S1). Primers mapping exclusively to *HYDIN* on chromosome 16 were designed (Table S2) which further resolved the next generation data from WES. The frameshift was found to be homozygous, and likely the causative allele. The splice site variant was heterozygous, but its pathogenicity is uncertain. Determination of segregation would help further resolve these variants, however, family members were not available. Ciliary biopsy of this individual was normal, as seen in other individuals with *HYDIN* mutations, further supporting the pathogenicity of the frameshift variant (Olbrich *et al.* 2012).

Individuals who did not have biallelic pathogenic mutations after WES were reanalyzed for rare missense variants of uncertain significance in the 32 known PCD genes. Five individuals from five families were found to harbor such VUS.

Individual 44 was found to have a homozygous missense c.1555G > C (p.A519P) variant in *DNAAF3* (Table 2) on WES, which was not reported in other PCD patients nor present in our control databases. This transversion resulted in an amino acid change which is predicted to be tolerated by SIFT and MutationTaster, and only probably damaging by Polyphen-2. *DNAAF3* mutations are associated with ODA + IDA defects, not seen in this patient. We concluded this variant was not likely contributing to this patient’s PCD phenotype and this case remained unsolved.

The four other individuals were found to have rare missense VUS predicted to be damaging, disease-causing, or deleterious by at least one of the *in silico* prediction programs, MutationTaster, PolyPhen-2 and SIFT. These individuals underwent targeted CNV analysis to ascertain a second hit (category C, Figure 1).

**Table 2:** Variants of uncertain significance in known PCD genes through CNV and WES analysis

Family	Patient	Ethnicity	Sex	Age at Dx	Situs Status	Ciliary EM	nNO nL/min	Gene	hg 19 Genomic coordinates	Transcript	Exon/ Intron	Base Changes	Predicted Effect	Segregation
Compound heterozygous, likely pathogenic (solved)														
114	9	White	M	4 yr	S	ODA+IDA*a*	13.4	*DYX1C1*	Chr15: 55727162	NM_130810.3	Ex 8	c.988C > T	p.R330W	Maternal
								*DYX1C1*	Chr15: 55729606-	NM_130810.3	Int 7	**Ex 7 2.1Kb del (6)**		Paternal
									55731727		Ex 7			
132	21	White	M	6 yr	I	Normal	4.9	*DNAH11*	Chr7: 21847620	NM_001277115.1	Ex 63	c.10285C > A	p.R3429S	Unknown
								*DNAH11*	Chr7: 21604615-	NM_001277115.1	Int 6	Ex7-14 32.29Kb dup		Unknown
									21636907		Int 14			
Homozygous VUS														
142	51	Sri Lankan Tamil	F	18 yr	S	Inconclusive	14.5	*DNAH11*	Chr7: 21639487	NM_001277115.1	Ex 15	c.2750A > T	p.E917V	Unknown
* *								*DNAH11*	Chr7: 21847621	NM_001277115.1	Ex 63	c.10286G > T	p.R3429L	Unknown
* 136*	44	Portuguese	M	8 yr	S	Normal	20.1	*DNAAF3*	Chr19: 55670702	NM_001256714.1	Ex 12	c.1555G > C	p.A519P	Unknown
*Single VUS*														
134	38	Hispanic	M	15 yr	S	Normal	17	*DNAH11*	Chr7: 21940666	NM_001277115.1	Ex 82	c.13345C > T	p.R4449C	Unknown

Genomic coordinates are approximate for copy number variations. Further clinical characteristics previously described (Kim *et al.* 2014). Previously described mutations are in **bold** (6) (Tarkar *et al.* 2013). Cases with two VUS, likely pathogenic were considered solved cases. PCD, primary ciliary dyskinesia; CNV, copy number variation; WES, whole-exome sequencing; Dx, diagnosis; EM, electron microscopy; M, male; S, situs solitus; I, situs inversus; ODA, outer dynein arms, IDA inner dynein arms; F, female; inconclusive = adequate sample inconclusive EM.

a Revised after pathology review.

Individual 9 was initially found to have a single variant in *DNAH5* (c.3890A > G; p.D1297G) that was predicted to be disease-causing by Poly-Phen, SIFT, and MutationTaster and ciliary ultrastructure was interpreted as ODA (Kim *et al.* 2014). However, array CGH analysis of all exons of *DNAH5* did not yield a second allele, prompting further review of WES data. In addition to the rare variant in *DNAH5*, a single rare missense variant in *DYX1C1* (c.988C > T; p.R330W) was found in WES data. This variant was not observed in control populations and was also predicted to be disease causing by SIFT and MutationTaster. Targeted CNV analysis by TaqMan copy number assay of a previously reported exon 7 deletion in *DYX1C1* was conducted and was found in this individual (Tarkar *et al.* 2013). Subsequent segregation analysis confirmed *trans* orientation of the rare missense variant and deletion. This prompted further pathologic review and revision of the initial ciliary EM to ODA + IDA consistent with other *DYX1C1* families (Table 2) (Tarkar *et al.* 2013), suggesting this is the causative gene in this family.

Individual 21 was found to have a rare missense VUS (c.10285C > A) in *DNAH11* on initial Sanger sequencing and WES confirmed this VUS. MutationTaster, PolyPhen-2 and SIFT algorithms predict the p.R3429S change to be deleterious, probably damaging and damaging respectively. Additionally, this is a highly conserved amino-acid residue residing in the conserved Helix 2 domain (Schwabe *et al.* 2008). Array CGH analysis on *DNAH11* was conducted and demonstrated a 32.29-kb duplication spanning exons 7−14 and is likely the causative second allele in this individual (Table 2).

Individual 51 was found to have two previously unreported homozygous missense variants in *DNAH11*, which also were detected through WES. One variant, c.10286G > T (p.R3429L) involves the same highly conserved amino-acid residue in the Helix 2 domain observed in individual 21. The other variant, c.2750A > T (p.E917V) also was predicted to be possibly damaging by Polyphen-2, deleterious by SIFT, and deleterious by MutationTaster. Subsequent array CGH of *DNAH11* analysis did not reveal a deletion or duplication and this case remained unsolved (Table 2).

Patient 38 was heterozygous in exon 82 of the *DNAH11* gene for an undocumented missense variant defined as c.13366C > T and predicted to result in p.R4456C substitution. The SIFT and PolyPhen-2 protein function algorithms predicts this change to be tolerated and benign, whereas the MutationTaster program indicates that this c.13366C > T variant is deleterious. However, *DNAH11* array CGH did not yield a second allele de-prioritizing this variant leaving this case unsolved (Table 2).

Through Sanger sequencing of the first 12 PCD genes, we found that five individuals who had a clinical diagnosis of PCD did not harbor a disease-causing mutation in a known PCD gene, nor did we find, using WES, a pathogenic mutation or rare missense VUS in any of the known PCD genes (Table S3). We suspect these patients either have a mutation not detectable by either technique or a novel PCD locus.

Overall, the combination of targeted CNV and WES analysis allowed for a molecular diagnosis in 55% of our unsolved families (11/20). Clinically significant CNVs were detected in 8.8% (4/45) of our total patient population, which is consistent with other autosomal-recessive disorders (Aradhya *et al.* 2012). Of the 45 families, Sanger sequencing solved 19 (42%) families whereas subsequent targeted CNV analysis with WES solved an additional 11, yielding an overall diagnostic rate of 30/45 (67%). Four further families were enrolled in other studies, such that in our cohort, 34/45 (76%) had a molecular diagnosis of PCD. As all variants detected by Sanger sequencing were covered in our WES data, if our molecular genetic approach was modified to WES followed by targeted CNV analysis, the overall diagnostic rate would approach 76%.

## Discussion

In genetically heterogeneous diseases such as PCD, a step-wise molecular genetic approach has been proposed based on ciliary ultrastructure and the prevalence of certain mutations. If genes are assayed in a cost-efficient, step-wise tiered fashion, only one gene may be assayed at one time, taking up to 4−6 months to complete, costing up to $12,000 USD. In addition, expert EM ciliary ultrastructure analysis may not be available to guide such tiered analysis. Newly developed next-generation sequencing (NGS) targeted panels are less costly, at $1900−$4200 USD, but do not include all 32 PCD genes (Prevention Genetics; Ambry Genetics, Aliso Viejo, CA; Center for Genomics and Transcriptomics, Tübingen, Germany). However, unlike tiered Sanger sequencing, NGS concurrently assays all genes. If a patient had previous genetic testing, these genes would be unnecessarily retested. Furthermore CNV data from NGS is unreliable and not possible with Sanger sequencing.

Here, we describe the utility of filtered WES data and targeted CNV analysis to circumvent the limitations of targeted Sanger and NGS panel sequencing in PCD. Because of the cost-efficiency of WES ($2−$5000 USD) (Neveling *et al.* 2013), we propose that WES could be conducted as a first-line molecular diagnostic test through the analysis of an *in silico* gene panel, followed by targeted CNV analysis. In addition to providing a definitive molecular diagnosis when biallelic pathogenic mutations are found, WES provides additional information on all PCD genes and aids in the interpretation of rare missense VUS. In the absence of convincing pathogenic mutations in all known PCD genes, rare missense VUS could result in loss of function and potentially be disease-causing prompting further targeted CNV analysis. CNV analysis should be targeted to the candidate PCD-associated gene on the basis of sequencing results. CNV analysis initially could begin with CNVs reported other PCD cohorts, followed by more extensive full exon analysis using array CGH or multiplex ligation-dependent probe amplification (Stuppia *et al.* 2012). Further studies are required to determine if the novel CNVs reported in our cohort are private mutations or common to other PCD patients. In addition, as CNV algorithms from WES data improve, they may replace other CNV analyses and provide a genome-wide CNV approach (Samarakoon *et al.* 2014). If a PCD case remains unsolved after WES and targeted CNV analysis, WES data can be further analyzed as novel PCD loci become published expanding the *in silico* panel in real-time, instead of expending further sequencing consumables. In our population, if WES was followed by targeted CNV analysis, 76% of patients with PCD would have had a molecular genetic diagnosis and WES should be considered the most cost-efficient molecular genetic technique in such genetically heterogeneous disorders.

## Supplementary Material

Supporting Information
