# Supplementary material for: Whole-Exome Sequencing and Targeted Copy Number Analysis in Primary Ciliary Dyskinesia
Source: G3 (Bethesda). 2015 Jul 2;5(8):1775–81. doi: 10.1534/g3.115.019851 (PMC4528333; doi:10.1534/g3.115.019851)
Supplement: Supporting Information [file supp_5_8_1775__index.html]

Whole-Exome Sequencing and Targeted Copy Number Analysis in Primary Ciliary Dyskinesia — Supporting Information 

# Whole-Exome Sequencing and Targeted Copy Number Analysis in Primary Ciliary Dyskinesia

## Supporting Information for Marshall *et al.*, 2015

**Files in this Data Supplement:**

- Supporting Information - File S1, Tables S1-S3, and Figure S1 (PDF, 172 KB)
- File S1 - Supplemental Material and Methods (PDF, 79 KB)
- Table S1 - Pathogenic mutations identified through other studies. (PDF, 92 KB)
- Table S2 - Primers sequences for Sanger validation. (PDF, 75 KB)
- Table S3 - Patients with no mutations in PCD genes through WES. (PDF, 88 KB)
- Figure S1 - Sanger sequencing of *HYDIN* variants c.13680-1G>T (A) and c.4866del; p.P1623Qfs\*20 (B) with chromatogram and *in silico* PCR sequence. (PDF, 101 KB)
