# Supplementary material for: Whole-Exome Sequencing and Targeted Copy Number Analysis in Primary Ciliary Dyskinesia
Source: G3 (Bethesda). 2015 Jul 2;5(8):1775–81. doi: 10.1534/g3.115.019851 (PMC4528333; doi:10.1534/g3.115.019851)
Supplement: Supporting Information [file supp_g3.115.019851_019851SI.pdf]

G3: Genes | Genomes | Genetics

WHOLE EXOME SEQUENCING AND TARGETED COPY NUMBER ANALYSIS IN PRIMARY CILIARY DYSKINESIA

Christian R Marshall[*,§], Stephen W Scherer[§,†], Maimoona A Zariwala[‡], Lynette Lau[*], Tara A Paton[§], Tracy Stockley[*], Rebekah K Jobling[**], Peter N Ray[*], Michael R Knowles[§§], FORGE Canada Consortium, GDMCC consortium, David A Hall[††,‡‡,‡‡‡], Sharon D Dell[***,§§§,†††,‡‡‡] and Raymond H Kim[**,‡‡,†††, ‡‡‡].

[*]Department of Pediatric Laboratory Medicine, The Hospital for Sick Children, Toronto, ON, Canada

[§]The Centre for Applied Genomics, Genetics and Genome Biology, The Hospital for Sick Children, Toronto, ON, Canada

[†]Department of Molecular Genetics and the McLaughlin Centre, University of Toronto, Toronto, ON, Canada

[‡]Department of Pathology and Laboratory Medicine, University of North Carolina School of Medicine, Chapel Hill, NC, USA

[**]Division of Clinical and Metabolic Genetics, The Hospital for Sick Children, Toronto, ON, Canada

[§§]Department of Medicine, University of North Carolina School of Medicine, Chapel Hill, NC, USA

[††]Division of Respirology, St. Michael's Hospital, Toronto, ON, Canada

[‡‡]Department of Medicine, University of Toronto, Toronto, ON, Canada

[***]Child Health Evaluative Sciences, The Hospital for Sick Children, ON, Canada

[§§§]Division of Respiratory Medicine, The Hospital for Sick Children, ON, Canada

[†††]Department of Pediatrics, University of Toronto, Toronto, ON, Canada

[‡‡‡]**These authors jointly directed this work, correspondence should be addressed to:**

Raymond H Kim, MD/PhD, FRCPC, FCCMG, FACMG
Division of Metabolic and Clinical Genetics, The Hospital for Sick Children,
555 University Ave, Toronto, ON, M5G 1X8.
Phone: 416-813-5338
Fax: 416-813-5345
raymond.kim@utoronto.ca

Sharon D. Dell, Beng, MD, FRCPC
Division of Respiratory Medicine, Room 4543, The Hospital for Sick Children,
555 University Ave, Toronto, ON, M5G 1X8.
Phone: 416-813-6248
Fax: 416-813-6246
sharon.dell@sickkids.ca

David A. Hall MD PhD FRCPC
Division of Respirology, St Michael's Hospital,
30 Bond Street, Toronto, ON, M5B 1W8
Phone: 416-846-6060 x 5516
Fax: 416-846-5649
hallda@smh.ca

DOI: 10.1534/g3.115.019851

File S1

Supplemental Material and Methods

Sanger sequencing

PCR primers targeting the variant region were designed using Primer3 on genomic DNA sequence masked for annotated SNPs. (Table S1). 50ng of genomic DNA for each sample was amplified and Sanger sequenced at the Hospital for Sick Children Molecular diagnostic lab, a CLIA-CAP certified laboratory, on a 3730XL DNA Analyzer (Life Technologies). For HYDIN, locus specific primers were designed (Table S2) and sequence was blatted to ensure only the chromosome 16 locus was amplified (Figure S1).

Copy Number Estimation of *DYX1C1* with Taqman copy number assay

Copy number estimation of exon 7 of *DYX1C1* was performed using the Taqman copy number probes Hs02618794_cn (chr.15:55,727,217), Hs05342683_cn (chr.15:55,729,606), and Hs02608276_cn (chr.15:55,731,727) (Life Technologies) using the manufacturer's recommended protocol. The assay was performed in quadruplicate on 10ng genomic DNA for each sample in a 96-well plate. The 10 µl reaction mix consisted of 2µl 2x Taqman Genotyping Master Mix (Life Technologies), 0.5 µl of 20X copy number assay, 0.5 µl TaqMan RNAse P Copy Number Reference Assay (Life Technologies, part 4403326), 2 µl water and 2 µl of 5ng/µl genomic DNA. Cycling conditions for the reaction were 95°C for 10 min, followed by 40 cycles of 95°C for 15 sec and 60°C for 1 min. Samples were analyzed using the ViiA™ 7 Real-Time PCR System (Life Technologies) and analyzed using CopyCaller Software (Life Technologies, PN 4412907).

Table S1 Pathogenic mutations identified through other studies

Family	Patient	Ethnicity	Sex	nNO nL/min	Ciliary EM	Gene	Exon	Base Changes	Predicted Effect	Segregation
118	35	Pakistani	F	23.4	ODA+IDA	*LRRC6*	5	c.630delG	p.W210Cfs*12 (2)	Unknown
118	37	Pakistani	M	36.5	Not done	*LRRC6*	5	c.630delG	p.W210Cfs*12 (2)	Unknown
110	15	Pakistani	F	10.8	ODA+IDA	*CCDC103*	3	c.461A>C	p.H154P (6)	Mother carrier
117	17	Somali	M	107.2	ODA+IDA	*SPAG1*	12	c.2542delG	p.D848I*fs10 (4)	Unknown
117	19	Somali	M	8.8	ODA+IDA	*SPAG1*	18	c.2542delG	p.D848I*fs10 (4)	Unknown
119	18	White	F	1.0	ODA+IDA	*SPAG1*	9	c.897_901del	p.K301* (4)	Paternal
							16	c.1993_1996del	p.L665* (4)	Maternal

nNo, nasal nitric oxide; F, female; M, male; Mo, months; ODA, outer dynein arms; IDA, inner dynein arms. Further clinical characteristics previously described (Kim et al. 2014). (2) (Zariwala et al. 2013); (4) (Knowles et al. 2013); (6) (Panizzi et al. 2012).

Table S2 Primers sequences for Sanger validation

Gene	Exon/Intron	Forward Primer	Reverse Primer
LRRC6	Ex 5	GGGCTGATTCACACTGCTAC	CAGAATTGTCACAAGCAATGG
SPAG1	Ex 12	GAGTTCTGCATGATCAGTCTGC	GAATTAGGGCGGTAGCAGTG
ZMYND10	Ex 1	GAGAACTGACGCTCCCCAAC	CCCGACTCAAGGACAATGAC
	Ex 3	GGGATATCAGGGTTGAGCTG	AGAGAAGGACAGGGCCTGAG
RSPH1	Ex 2	TCTAGCCCAGGCGTTGTTAC	TGTCAGTATTCACAGACAAGTTCAG
	Ex 4	CACAACACATCTGCCTTTGC	ACTTGCACAGAAAGGCATCC
ARMC4	Ex 3	AATGATCCTCCCACCCTTTC	CTAGGCTTGCTGGTAAGTTTGAG
	Ex 18	TCACAGCTGTATTGTTACTTTCTGC	AATCCAGGTTTCTGGACTGC
CCNO	Ex 3	TCCTGAAGCCTTCTCTGTGG	CTGCACAAGCTGCACTTCAC
HYDIN	Ex 32	GACAGTACTGATGGGTTCCTTGA	GGAGTTAACGTTGTTTCTAGTCTCCA
	Int 79	GCAGCAGCTACTAACCTCTTTTACC	AGGGAGGTGAACCTCAGCCT
DNAAF3	Ex 12	CTTCTCATCCCTGAGCTTGG	CCTCTGAGAGTGAACCTGGAG
DYX1C1	Ex 8	GATTCAGTGCAGGCAACGTTT	GCAATATAGCGAGACCCACT

Table S3 Patients with no mutations in PCD genes through WES

Family	Patient	Sex	nNO nL/min	Ciliary EM	Situs Status	Ethnicity
121	30	M	15.3	IDA+CP	S	Portuguese
133	25	M	13.8	Normal	S	White
131	20	M	99.2	Inconclusive	I	White
139	48	F	56.2	Inconclusive	S	Pakistani
143	53	F	18.4	Inconclusive	S	White

nNo, nasal nitric oxide; F, female; M, male; Yr, years; S, situs solitus; I, situs inversus;IDA, inner dynein arms; CP, central pair; inconclusive=adequate sample inconclusive TEM

A. chr16: 70,866,971 C>A (c.13680-1G>T)

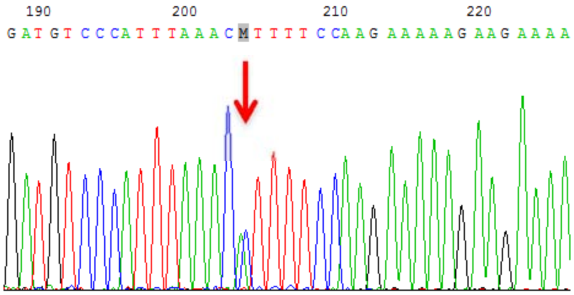

>chr16:70866736-70867075 (340bp)
GCAGCAGCTACTAACCTCTTTTACCgcaggtggtcccacgcagactccagacagggttagactcagagga
ctgcctccctggatgtagcagagaatgttttacaaaggctctcctttcccacctcggtgggatggtagg
tcacttcaaaagaaacctccatgcctgaggtaatatagccttcttctgggctaatggagaaatgaggctc
aaatttttgatgtcccatttaaacctttccaagaaaaagaagaaaagaaagagagtttatggatgttg
taagggtgtcctcaatgctactgagaacaagagactgcctAGGCTGAGGTTCACCTCCCT

B. chr16:71,008,480 delA (c.4866del; p.P1623Qfs*20)

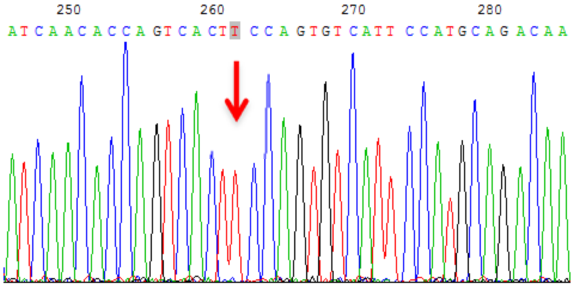

>chr16:71008367-71008741 (375bp)
GACAGTACTGATGGGTTCCTTGAgagaaatgaccaagtgcaataggaaagggtctccccagcctgggtac
ctgtctcatgaaggacacgcttgtctgcatggaatgacactggaaagtgactggtgttgatgatcttgat
gatgtgggttcggacttcgccaaggatgatgtagccaaagtccaggatgtactctggtagctggattctg
aaatggttaatagaccttactagtcactttcaaacctggaaggctacttttccaaaagtgagtgagtctg
atttttttttctacctcctgtgcacagttttattctccagagtggagaacaacaaatggctacccaaaT
GGAGACTAGAAACAACGTTAACTCC

Figure S1 Sanger sequencing of *HYDIN* variants c.13680-1G>T (A) and c.4866del; p.P1623Qfs*20 (B) with chromatogram and *in silico* PCR sequence. Red Arrow depicts variant in the Sanger chromatogram and variant position is highlighted red in the amplicon sequence below. Capital letters are the locus specific primers (Table S2) and variants highlighted in yellow depict nucleotide sequence specific to chromosome 16.
