# Supplementary material for: Whole-Exome Sequencing and Targeted Copy Number Analysis in Primary Ciliary Dyskinesia
Source: G3 (Bethesda). 2015 Jul 2;5(8):1775–81. doi: 10.1534/g3.115.019851 (PMC4528333; doi:10.1534/g3.115.019851)
Supplement: Supporting Information [file supp_g3.115.019851_FileS1.pdf]

File S1

Supplemental Material and Methods

Sanger sequencing

PCR primers targeting the variant region were designed using Primer3 on genomic DNA sequence masked for annotated SNPs. (Table S1). 50ng of genomic DNA for each sample was amplified and Sanger sequenced at the Hospital for Sick Children Molecular diagnostic lab, a CLIA-CAP certified laboratory, on a 3730XL DNA Analyzer (Life Technologies). For HYDIN, locus specific primers were designed (Table S2) and sequence was blatted to ensure only the chromosome 16 locus was amplified (Figure S1).

Copy Number Estimation of *DYX1C1* with Taqman copy number assay

Copy number estimation of exon 7 of *DYX1C1* was performed using the Taqman copy number probes Hs02618794_cn (chr.15:55,727,217), Hs05342683_cn (chr.15:55,729,606), and Hs02608276_cn (chr.15:55,731,727) (Life Technologies) using the manufacturer's recommended protocol. The assay was performed in quadruplicate on 10ng genomic DNA for each sample in a 96-well plate. The 10 µl reaction mix consisted of 2µl 2x Taqman Genotyping Master Mix (Life Technologies), 0.5 µl of 20X copy number assay, 0.5 µl TaqMan RNAse P Copy Number Reference Assay (Life Technologies, part 4403326), 2 µl water and 2 µl of 5ng/µl genomic DNA. Cycling conditions for the reaction were 95°C for 10 min, followed by 40 cycles of 95°C for 15 sec and 60°C for 1 min. Samples were analyzed using the ViiA™ 7 Real-Time PCR System (Life Technologies) and analyzed using CopyCaller Software (Life Technologies, PN 4412907).
