# Supplementary material for: Whole-Exome Sequencing and Targeted Copy Number Analysis in Primary Ciliary Dyskinesia
Source: G3 (Bethesda). 2015 Jul 2;5(8):1775–81. doi: 10.1534/g3.115.019851 (PMC4528333; doi:10.1534/g3.115.019851)
Supplement: Supporting Information [file supp_g3.115.019851_TableS1.pdf]

## Table S1 Pathogenic mutations identified through other studies

| Family | Patient | Ethnicity | Sex | nNO nL/min | Ciliary EM | Gene | Exon | Base Changes | Predicted Effect | Segregation |
|---|---|---|---|---|---|---|---|---|---|---|
| 118 | 35 | Pakistani | F | 23.4 | ODA+IDA | *LRRC6* | 5 | c.630delG | p.W210Cfs*12 (2) | Unknown |
| 118 | 37 | Pakistani | M | 36.5 | Not done | *LRRC6* | 5 | c.630delG | p.W210Cfs*12 (2) | Unknown |
| 110 | 15 | Pakistani | F | 10.8 | ODA+IDA | *CCDC103* | 3 | c.461A>C | p.H154P (6) | Mother carrier |
| 117 | 17 | Somali | M | 107.2 | ODA+IDA | *SPAG1* | 12 | c.2542delG | p.D848I*fs10 (4) | Unknown |
| 117 | 19 | Somali | M | 8.8 | ODA+IDA | *SPAG1* | 18 | c.2542delG | p.D848I*fs10 (4) | Unknown |
| 119 | 18 | White | F | 1.0 | ODA+IDA | *SPAG1* | 9 | c.897_901del | p.K301* (4) | Paternal |
| | | | | | | | 16 | c.1993_1996del | p.L665* (4) | Maternal |

nNo, nasal nitric oxide; F, female; M, male; Mo, months; ODA, outer dynein arms; IDA, inner dynein arms. Further clinical characteristics previously described (Kim et al. 2014). (2) (Zariwala et al. 2013); (4) (Knowles et al. 2013); (6) (Panizzi et al. 2012).
