# Supplementary material for: Whole-Exome Sequencing and Targeted Copy Number Analysis in Primary Ciliary Dyskinesia
Source: G3 (Bethesda). 2015 Jul 2;5(8):1775–81. doi: 10.1534/g3.115.019851 (PMC4528333; doi:10.1534/g3.115.019851)
Supplement: Supporting Information [file supp_g3.115.019851_TableS2.pdf]

Table S2   Primers sequences for Sanger validation

Gene	Exon/Intron	Forward Primer	Reverse Primer
LRRC6	Ex 5	GGGCTGATTCACACTGCTAC	CAGAATTGTCACAAGCAATGG
SPAG1	Ex 12	GAGTTCTGCATGATCAGTCTGC	GAATTAGGGCGGTAGCAGTG
ZMYND10	Ex 1	GAGAACTGACGCTCCCCAAC	CCCGACTCAAGGACAATGAC
	Ex 3	GGGATATCAGGGTTGAGCTG	AGAGAAGGACAGGGCCTGAG
RSPH1	Ex 2	TCTAGCCCAGGCGTTGTTAC	TGTCAGTATTCACAGACAAGTTCAG
	Ex 4	CACAACACATCTGCCTTTGC	ACTTGCACAGAAAGGCATCC
ARMC4	Ex 3	AATGATCCTCCCACCCTTTC	CTAGGCTTGCTGGTAAGTTTGAG
	Ex 18	TCACAGCTGTATTGTTACTTTCTGC	AATCCAGGTTTCTGGACTGC
CCNO	Ex 3	TCCTGAAGCCTTCTCTGTGG	CTGCACAAGCTGCACTTCAC
HYDIN	Ex 32	GACAGTACTGATGGGTTCCTTGA	GGAGTTAACGTTGTTTCTAGTCTCCA
	Int 79	GCAGCAGCTACTAACCTCTTTTACC	AGGGAGGTGAACCTCAGCCT
DNAAF3	Ex 12	CTTCTCATCCCTGAGCTTGG	CCTCTGAGAGTGAACCTGGAG
DYX1C1	Ex 8	GATTCAGTGCAGGCAACGTTT	GCAATATAGCGAGACCCACT
