# Supplementary material for: Whole-Exome Sequencing and Targeted Copy Number Analysis in Primary Ciliary Dyskinesia
Source: G3 (Bethesda). 2015 Jul 2;5(8):1775–81. doi: 10.1534/g3.115.019851 (PMC4528333; doi:10.1534/g3.115.019851)
Supplement: Supporting Information [file supp_g3.115.019851_TableS3.pdf]

Table S3 Patients with no mutations in PCD genes through WES

Family	Patient	Sex	nNO nL/min	Ciliary EM	Situs Status	Ethnicity
121	30	M	15.3	IDA+CP	S	Portuguese
133	25	M	13.8	Normal	S	White
131	20	M	99.2	Inconclusive	I	White
139	48	F	56.2	Inconclusive	S	Pakistani
143	53	F	18.4	Inconclusive	S	White

nNo, nasal nitric oxide; F, female; M, male; Yr, years; S, situs solitus; I, situs inversus;IDA, inner dynein arms; CP, central pair; inconclusive=adequate sample inconclusive TEM
