# Supplementary material for: Whole-Exome Sequencing and Targeted Copy Number Analysis in Primary Ciliary Dyskinesia
Source: G3 (Bethesda). 2015 Jul 2;5(8):1775–81. doi: 10.1534/g3.115.019851 (PMC4528333; doi:10.1534/g3.115.019851)
Supplement: Supporting Information [file supp_g3.115.019851_FigureS1.pdf]

A. chr16: 70,866,971 C>A (c.13680-1G>T)

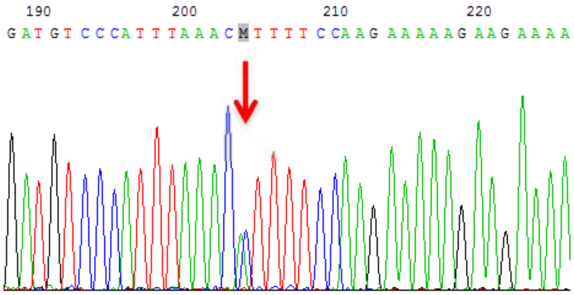

>chr16:70866736-70867075 (340bp)
GCAGCAGCTACTAACCTCTTTTACCgcaggtggtcccacgcagactccagacagggttagactcagagga
ctgcctccctggatgtagcagagaatgttttacaaaggctctcctttcccacctcggtgggatggtagg
tcacttcaaaagaaacctccatgcctgaggtaatatagccttcttctgggctaatggagaaatgaggctc
aaatttttgatgtcccatttaaacctttccaagaaaaagaagaaaagaaagagagtttatggatgttg
taagggtgtcctcaatgctactgagaacaagagactgcctAGGCTGAGGTTCACCTCCCT

B. chr16:71,008,480 delA (c.4866del; p.P1623Qfs*20)

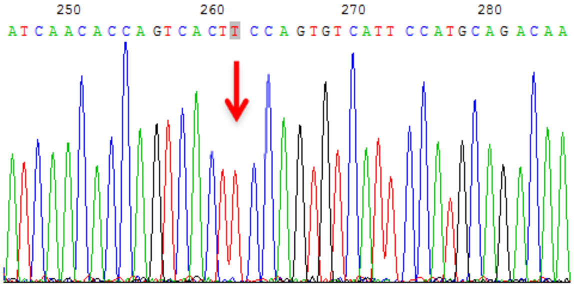

>chr16:71008367-71008741 (375bp)
GACAGTACTGATGGGTTCCTTGAgagaaatgaccaagtgcaataggaaagggtctccccagcctgggtac
ctgtctcatgaaggacacgcttgtctgcatggaatgacactggaaagtgactggtgttgatgatcttgat
gatgtgggttcggacttcgccaaggatgatgtagccaaagtccaggatgtactctggtagctggattctg
aaatggttaatagaccttactagtcactttcaaacctggaaggctacttttccaaaagtgagtgagtctg
atttttttttctacctcctgtgcacagttttattctccagagtggagaacaacaaatggctacccaaaT
GGAGACTAGAAACAACGTTAACTCC

Figure S1 Sanger sequencing of *HYDIN* variants c.13680-1G>T (A) and c.4866del; p.P1623Qfs*20 (B) with chromatogram and *in silico* PCR sequence. Red Arrow depicts variant in the Sanger chromatogram and variant position is highlighted red in the amplicon sequence below. Capital letters are the locus specific primers (Table S2) and variants highlighted in yellow depict nucleotide sequence specific to chromosome 16.
